# Arsenic-induced Histological Alterations in Various Organs of Mice

**DOI:** 10.4172/2157-7099.1000323

**Published:** 2015-04-24

**Authors:** Abu Shadat Mohammod Noman, Sayada Dilruba, Nayan Chandra Mohanto, Lutfur Rahman, Zohora Khatun, Wahiduzzaman Riad, Abdullah Al Mamun, Shahnur Alam, Sharmin Aktar, Srikanta Chowdhury, Zahangir Alam Saud, Zillur Rahman, Khaled Hossain, Azizul Haque

**Affiliations:** 1 Department of Biochemistry and Molecular Biology, University of Chittagong, Chittagong, Bangladesh; 2 Department of Biochemistry and Molecular Biology, Rajshahi University, Rajshahi, Bangladesh; 3 Department of Pathology, Chittagong Medical College (CMC), Chittagong, Bangladesh; 4 Department of Microbiology and Immunology, Medical University of South Carolina, USA

**Keywords:** Arsenic, Histopathology, Mice organs

## Abstract

Deposition of arsenic in mice through groundwater is well documented but little is known about the histological changes of organs by the metalloid. Present study was designed to evaluate arsenic-induced histological alterations in kidney, liver, thoracic artery and brain of mice which are not well documented yet. Swiss albino male mice were divided into 2 groups and treated as follows: Group 1: control, 2: arsenic (sodium arsenite at 10 mg/kg b.w. orally for 8 wks). Group 2 showed marked degenerative changes in kidney, liver, thoracic artery, and brain whereas Group 1 did not reveal any abnormalities on histopathology. We therefore concluded that arsenic induces histological alterations in the tested organs.

## Introduction

Around 200 million people (NRC 2001) worldwide are at risk from health effects associated with high concentrations of arsenic in their drinking water [1]. Unfortunately, 75 millions of those people are living in Bangladesh. Hence, in the current century, Bangladesh is under a big threat of arsenic disaster. Humans are chronically exposed to arsenic, which is present in food, water, soil, and air. Chronic exposure to inorganic arsenic (iAs) that occurs as a natural contaminant in drinking water is associated with the development of skin cancer [2,3] and other severe health problems such as diabetes, liver, kidney, CNS disorders [4] and also causes many other toxic effects [5,6]. Recently, a series of animal and human epidemiological studies have indicated an association between As exposure and adverse reproductive and developmental outcomes [7–9]. Several line of studies [10,11] have indicated tissue architecture change in heart and hepatic organs by sodium arsenite (Sa). Exposure of Sa showed haemorrhages in myocardium, and degeneration and separation of muscle bundles. However, histopathological examination of the lungs after arsenic exposure showed a normal alveoli spaces and with normal alveoli cell [12]. Deposition of high concentrations of arsenic in the liver, kidney, lungs, hair and nails have been well reported [6]. Epidemiological studies have shown association between chronic arsenic exposure and liver disease and kidney failure [6]. The relationship between chronic arsenic exposure and the development of specific target organ toxicity is not completely understood. Moreover, the risk involvement of this metal on some organs like thoracic artery and brain is poorly understood. Understanding the organ specific histological effect of arsenic is necessarily important to know the details mechanism of arsenic mediated toxicity in mammals. Organ specific histological evaluation is currently the gold standard to determine the degree of organ injury during chronic metal exposure. Interestingly, organ function markers alter during histological degeneration. However, still there is no report describes the histological effect of arsenic on some organs such as artery, and brain where as other organs like kidney and liver are not well defined. This study was designed to evaluate the histological changes by chronic arsenic exposure on some tissue architecture in mice. A better understanding of the effect of arsenic at target organs with an emphasis on observation of tissue architecture at critical sites will aid in defining a mode(s) of action for arsenic-induced toxicity in mammals and reduce the uncertainty in the risk assessment for this metalloid.

## Material and Methods

### Animals and housing conditions

Swiss albino male mice (6 weeks of age) of average body weight (30 gm) were purchased from Animal Division of International Center for Diarrhoeal Disease Research, Bangladesh (ICDDR, B). The mice were randomly selected and kept in plastic cages with wood-cobe bedding (5 mice/cage). After five days of acclimation, mice were divided into two groups namely control and sodium arsenite (Sa) induced mice. They were maintained with 12-h:12-h dark light cycle with available supply of distilled water and feed. Sa was given to the mice with water (10 mg/kg body weight/day). The amounts of water consumed were recoded every day. Ethical approval for the study was duly obtained from the ethical committee of Faculty of Biological Sciences, University of Rajshahi, Bangladesh.

### Chemical and dosing

Sa(NaAsO_2_; Cat. No.30110, BDH (England) was dissolved in distilled water, and served as drinking water for mice. The dose levels of Sa, used in the present study, was set as: 2 ml of water (150 mg/L) per day; was reported to be 10 mg/Kg b.w. Same dose level was used in a series of studies [13,14]. Control mice were maintained with available supply of distilled water and normal mice feed. These two different groups of mice were maintained for 8 weeks. All these procedures and experiments using mice were undertaken following the ethical issues set by the Faculty of Biological Sciences, University of Rajshahi, Bangladesh.

### Histopathological study

At the end of 16 weeks, mice were euthanized and kidney, liver, thoracic artery and brain were collected in 10% buffered formalin solution, passed through ascending series of ethanol baths, cleared in toluene and embedded in paraffin. Tissues were sectioned at 4 μm and stained with Haematoxylin and Eosin (H&E). The sections were examined by light microscope.

### Sample collection and assessment of serum

Blood specimens were collected from the thoracic artery of the mice after anaesthetization with diethyl ether. For coagulation, blood was kept about 20 minutes at room temperature. After centrifugation at 1600 g for 15 minutes at 4°C, serum was drawn off and stored at −80°C until the experiments were performed.

### Liver and kidney function tests

The analyzer (CHEM-5 V3, Erba, Mannheim, Germany) were used for the measurement of serum indices by using commercially available kits according to the manufacture’s protocol. The level of blood urea nitrogen (BUN) and serum glutamate-pyruvate transaminase (SGPT) were measured by the kits from Human, Germany. All samples were analyzed in triplicate and then mean values were taken.

### Statistical analysis

Statistical analyses were performed with SPSS for windows, version 15.0 (SPSS, Chicago, IL). Data are expressed as mean ± SD or mean ± SE. Differences between the body weights and serum indices of different groups of mice were analyzed by using t-test.

## Results

**Figure 1:**
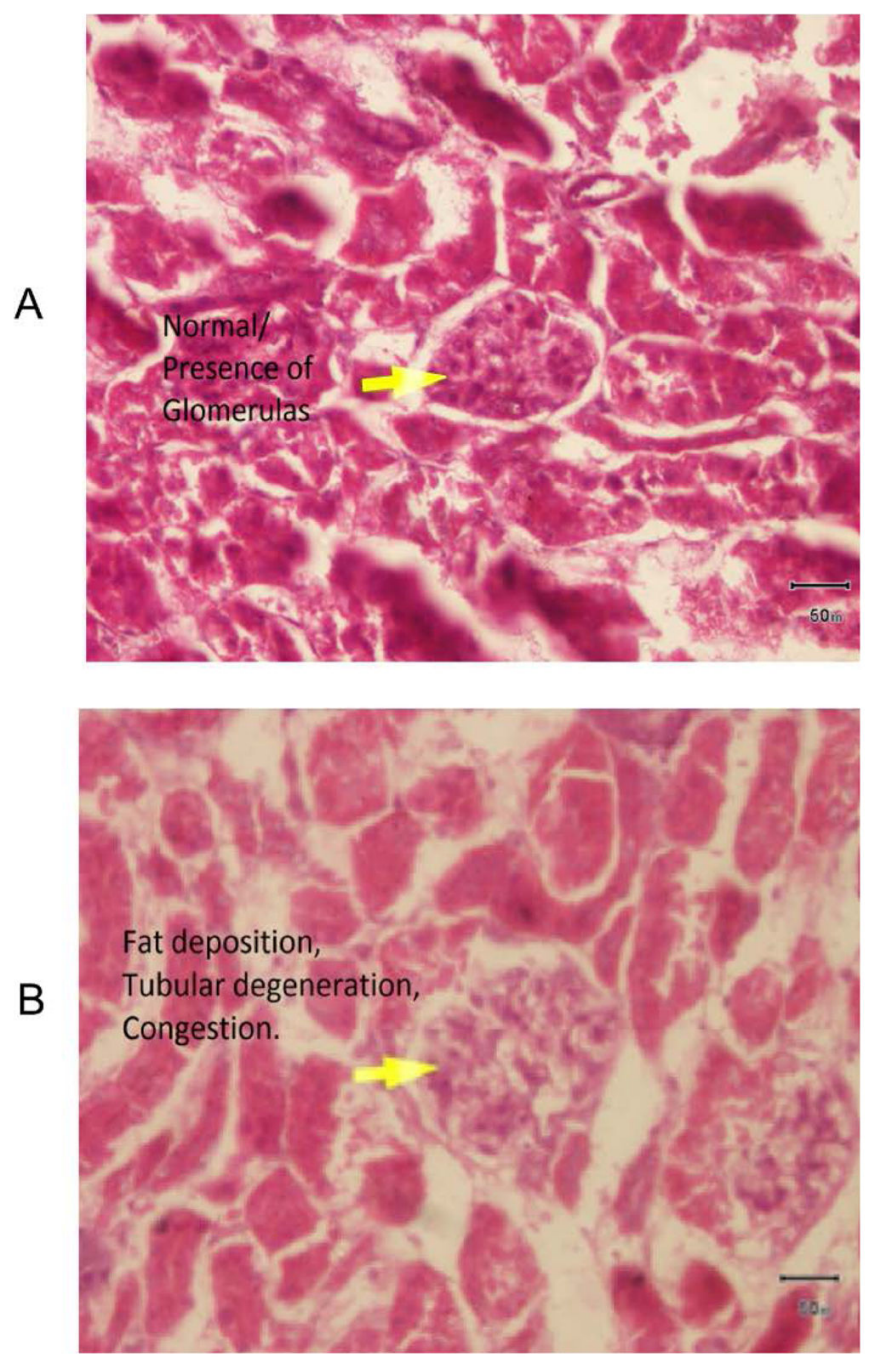
A) Photomicrograph of kidney showing normal architecture H&E X200 Group 1, B) Photomicrograph of kidney showing fat bodies, tubular degeneration, intratubular degeneration, and congestion, H&E 200X Group 2.

**Figure 2:**
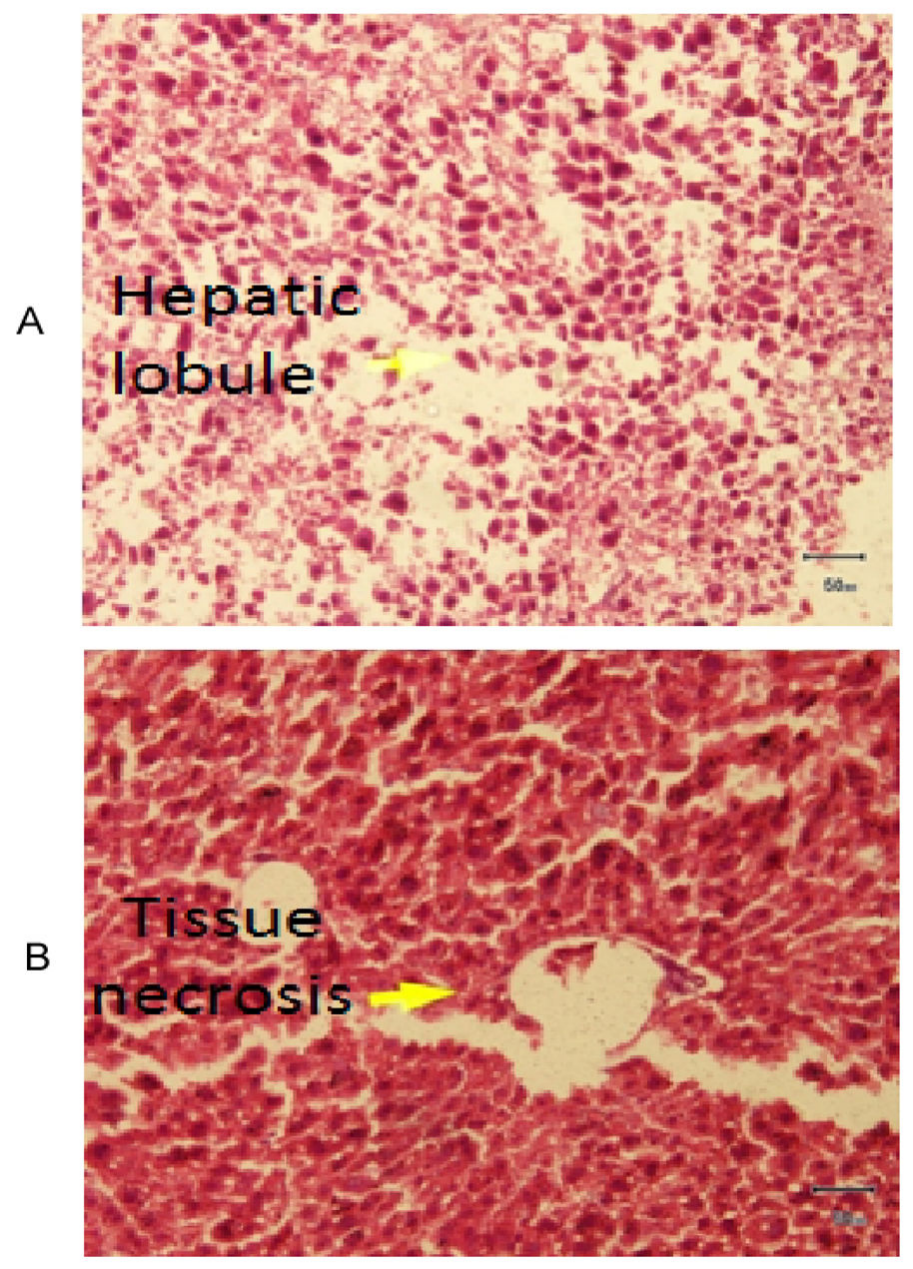
A). Photomicrograph of liver showing normal architecture H&E 200X Group 1, B). Photomicrograph of liver showing necrosis, H&E 200X Group 2.

**Figure 3:**
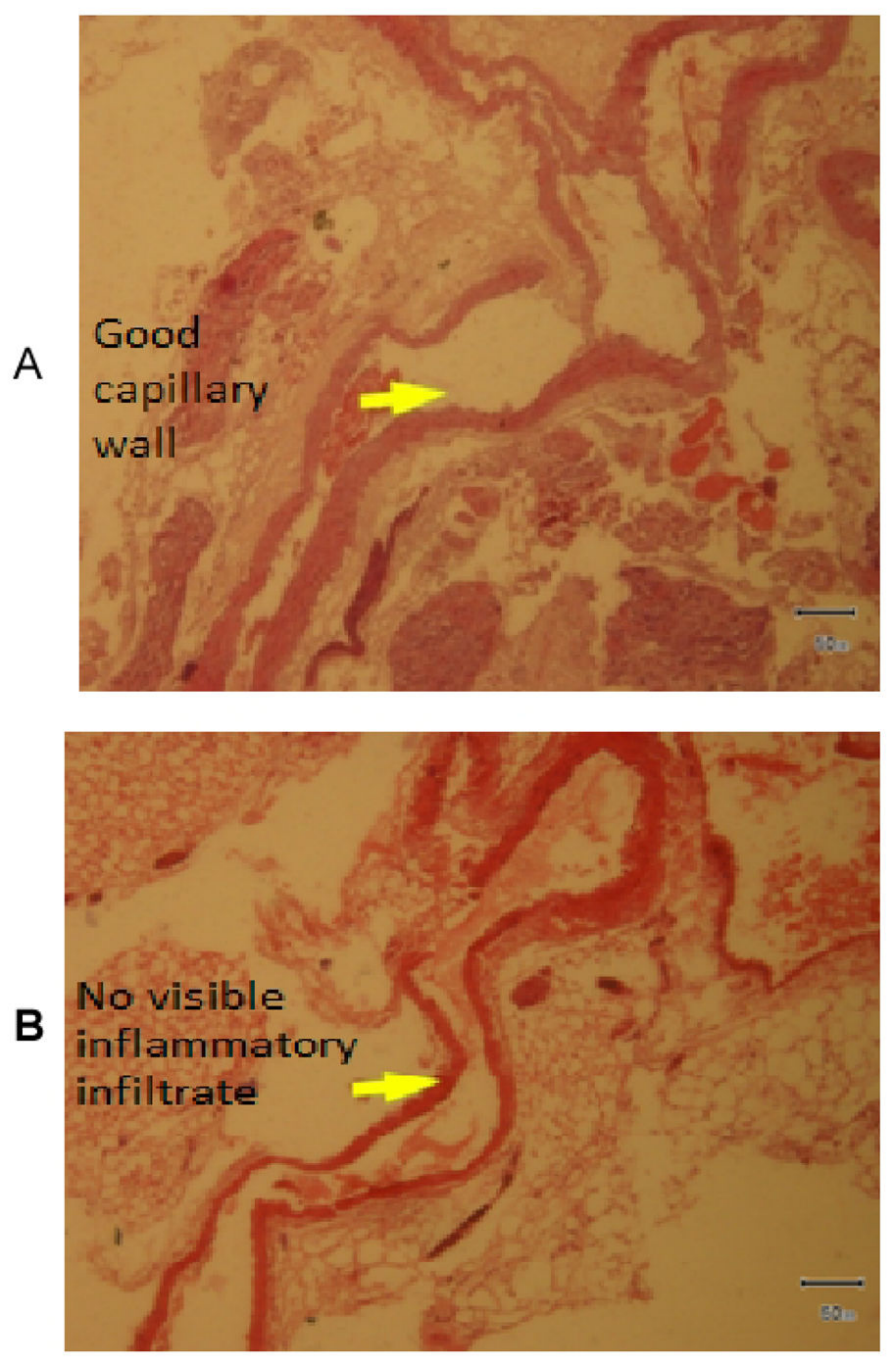
A). Photomicrograph of thoracic artery showing normal architecture H&E 200X Group 1, B). Photomicrograph of thoracic artery showing capillary wall was destructed, H&E 200X Group 2.

**Figure 4:**
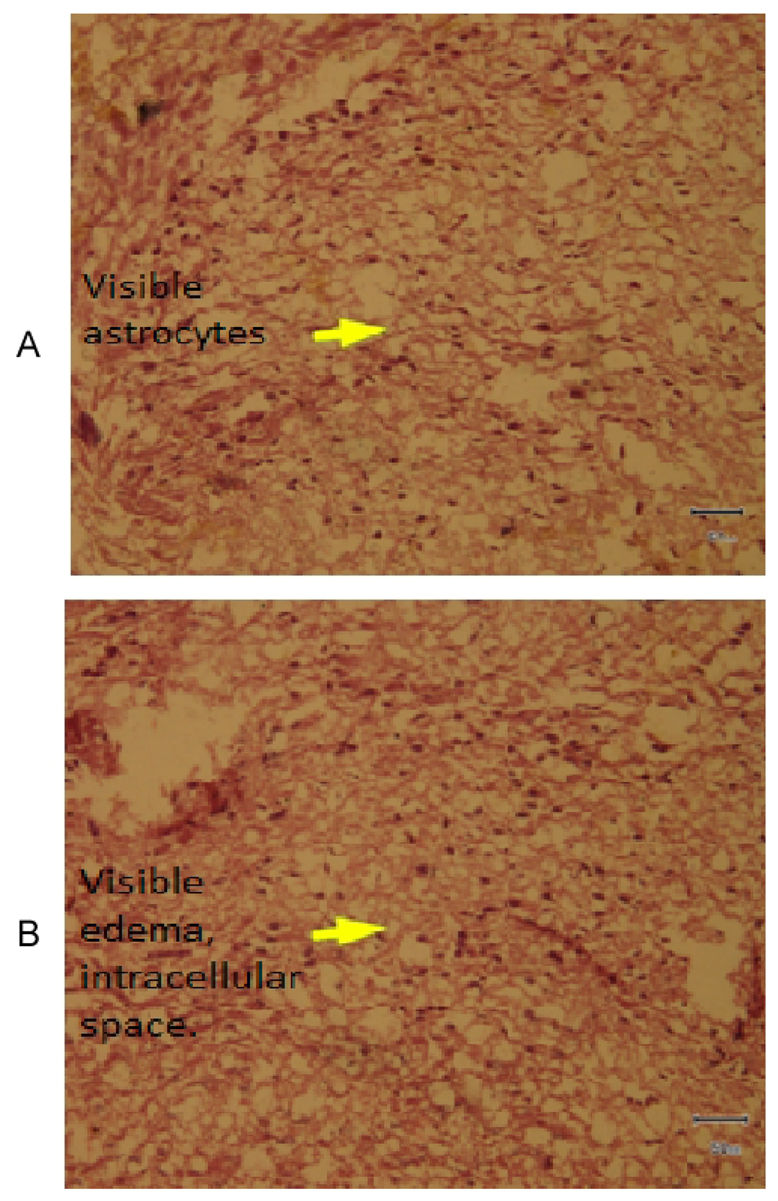
A). Photomicrograph of brain showing normal architecture H&E X200 Group 1, B). Photomicrograph of brain showing edema, intracellular space, edematous change, H&E 200X Group 2.

**Figure 5:**
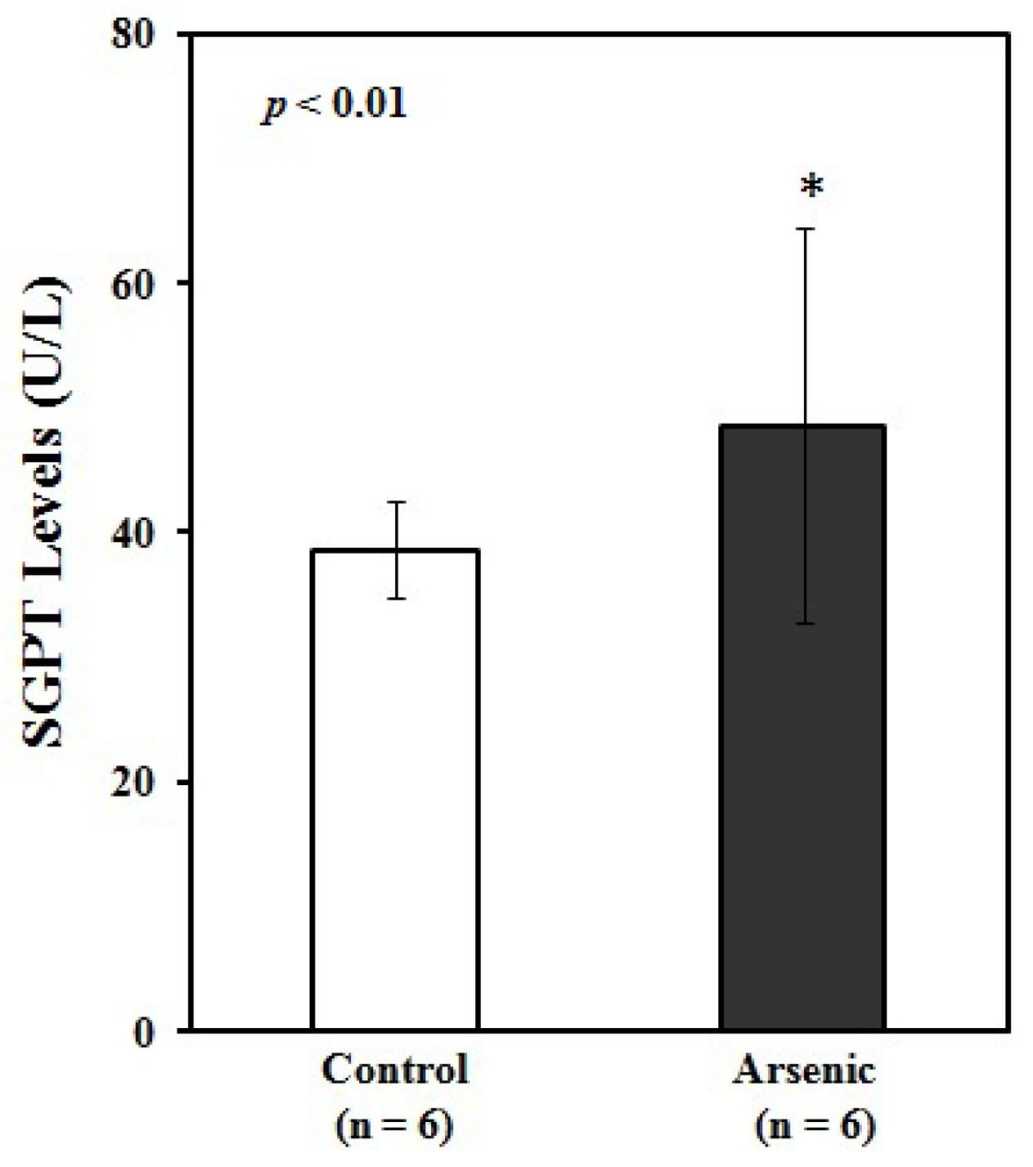
Comparison of serum glutamate-pyruvate transaminase (SGPT) levels (mean ± SE) between control and arsenic-treated mice. Blank and black bar represent the control and arsenic-treated mice, respectively. *Significantly different from control group at *p*<0.01. *p* value was from Independent sample t-test.

**Figure 6:**
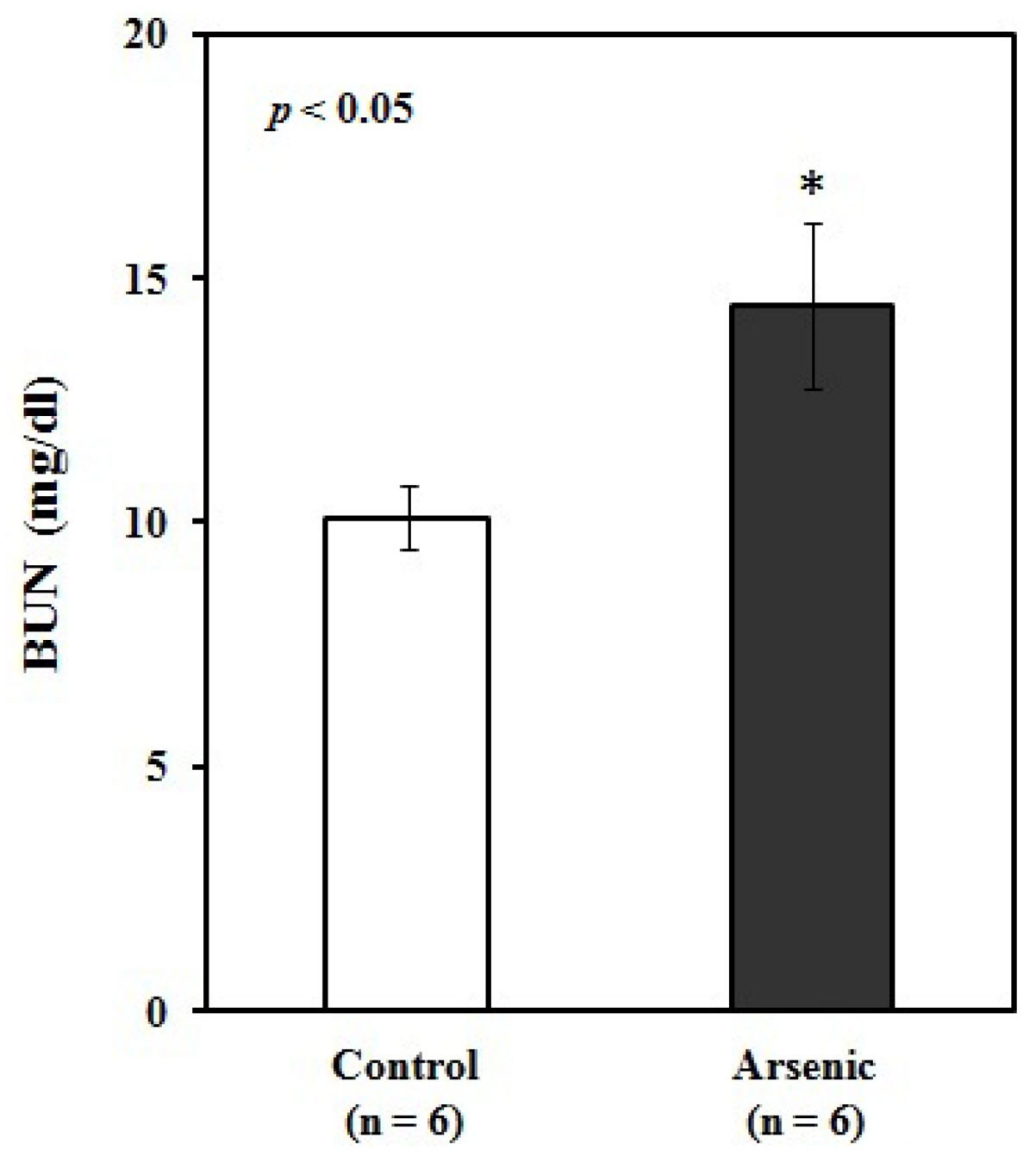
Comparison of blood urea nitrogen (BUN) levels (mean ± SE) between control and arsenic-treated mice. Blank and black bar represent the control and arsenic-treated mice, respectively. *Significantly different from control group at *p*<0.05. *p* value was from Independent sample t-test.

To understand the arsenic induced tissue alterations, we have performed histopathology for four types of tissue. We have observed fat bodies, tubular degeneration, intratubular degeneration, congestion in arsenic treated mice kidney tissue. In contrast, tissue architecture, glomerulas were normal in control kidney tissue (Figure 1). Heavy metals are prominent to necrosis in tissue especially in liver. We have also found some necrosis in Sa exposed liver tissue (Figure 2). In control liver tissue, hepatic lobules were intact. There were some alterations of morphological structure in arsenic exposed mice thoracic artery than normal tissue. In control artery, we have found good capillary wall. On the other hand, capillary wall was destructed in group 2, but there were no visible inflammatory infiltrate (Figure 3). We have also checked the architecture of brain tissue by histopathology. We have found well arrangement of tissue in normal brain tissue, whereas edema, intracellular space, edematous changes have seen in arsenic exposed brain tissue (Figure 4). The degree of serum SGPT and BUN can predict level of histological damage in liver and kidney, respectively. We therefore, checked the level of these biochemical parameters to further support the findings described above. Serum SGPT level was found to be significantly increased only in As exposure group as compared to the control group (Figure 5). Next, the level of BUN was also significantly increased in arsenic exposure group (Figure 6).

## Discussion

Increased oxidative stress in tissue due to arsenic exposure is seemed to be the major cause for arsenic-induced toxicity in mice. Arsenic mediated oxidative stresses are indicated for changing the organ degeneration during exposure. Arsenic concentrates in the kidney during its urinary elimination that affects the function of proximal convoluted tubules [15]. We have observed the degenerative changes in kidney tissue in arsenic treated mice (Figure 1). These findings may be justified by a recent observation which has shown hyperplasia in the bladder epithelium in mice treated with Sa in the diet [16]. Further we have observed the elevated level of BUN in arsenic exposed mice sample (Figure 6). BUN test is primarily used to evaluate kidney function in a wide range of circumstances, to monitor people with acute or chronic kidney dysfunction. We therefore interlinked between kidney tissue degeneration and elevated level of BUN, we have observed in our study. Earlier, another kidney function marker urinary NAG (N-acetyl-beta-glucosaminidase) was increased during arsenic exposure which presents a significant adverse impact on the kidney function in arsenic endemic areas [17]. We have also shown the arsenic exposure-related elevation of plasma uric acid (PUA) levels may be implicated in arsenic-induced cardiovascular diseases (CVDs). These series of reports support our current findings on the effect of arsenic on mice kidney. Kidney is an organ that is rich in phospholipids and leads to the oxidative degradation of phospholipids. We, therefore, hypothesized that arsenic-induced lipid peroxidation in kidney induces oxidative damage leading to functional deterioration.

There was a strong dose-response relationship between systemic arterial disease and cumulative arsenic exposure [18]. Pulmonary trunk and branch dilatation in chronic arsenicosis is a frequent abnormality seen in chest of arsenicosis patients. More recently, the association between low-level arsenic exposure and carotid artery intimal-medial thickness (IMT) is reported in human [19]. Although, concentration is given to establish the association between carotid arterial disease and chronic arsenic exposure, little is known on its effect on thoracic arterial system. This is the first time we have observed the arsenic mediated tissue degeneration in thoracic artery (Figure 3). We have observed the destruction of capillary wall of thoracic artery but no visible inflammatory infiltrate was seen.

Earlier, inorganic arsenicals have been reported to be accumulated in brain astrocytes [20]. Arsenic can disturb the mitosis of granule cells and interfere with the normal development of mice cerebellum [21]. Moreover, exposure of As with Cd and Pd impairs myelin and axon development in rat brain [22]. Early life exposure of arsenic develops neurotoxicity which disrupts normal behavioral pattern in human [23] also causes brain dopaminergic alterations in rats [24]. Despite the line of reports on arsenic mediated brain disorders, none has evaluated the histological changes in brain by arsenic. In the current report, we have found edema, intracellular space, as well as edematous change in arsenic exposed mice brain tissue (Figure 4). Chronic arsenic exposure induces oxidative damage in brain of rats [25]. We, therefore, assume that the brain architectural alteration is due to the oxidative stress mediated by Sa.

Liver has long been identified as a target organ of arsenic exposure. Because of its unique metabolic functions and related to the gastrointestinal tract, liver is an important target of toxicity to xenobiotics. The sections of liver in Sa group showed moderate degeneration and necrosis in hepatic parenchyma with mild to moderate fatty change where as control did not reveal any lesions of pathological significance (Figure 2). Earlier, arsenic mediated hepatocytic degeneration was characterized by vacuolar degeneration followed by hepatic necrosis observed in case of rat [11]. Further, we have observed increased SGPT level in arsenic exposed mice (Figure 5). Earlier, we and others have observed the elevated level of serum hepatic enzymes, alkaline phosphatase (ALP), aspartate transaminase (AST) and alanine transaminase (ALT) used for the liver function tests (LFTs) in the individuals exposed to arsenic [26–28]. Exposure of mice to arsenic in drinking water causes elevation of liver enzymes in plasma [29]. Like other toxic elements Sa primarily increased the generation of free radical species and cause an imbalance between pro-oxidation and antioxidant homeostasis in liver system as a result causes hepatic degeneration.

Arsenic mediated oxidative stress is associated with expression of antioxidant genes [30]. Moreover, arsenic-induced pathological changes may be caused by oxidative DNA damage other than nitrative DNA damage [21]. Oxidative stress through chronic arsenic exposure is associated with methyl insufficiency and loss of DNA methylation in animals [31–34] may be reason for the histological changes. We therefore believe that oxidative stress is associated with tissue architectural change in arsenicosis. The results of present study are in agreement with previous observation [35]. However, further investigation is necessarily important to know the details mechanism of Sa mediated changes in tissue architecture to better understand the mode of arsenic mediated toxicity.

## References

[1] Ravenscroft P, Brammer H, Richards KS (2009). Arsenic Pollution: A Global Synthesis.

[2] Tseng WP, Chu HM, How SW, Fong JM, Lin CS (1968). Prevalence of skin cancer in an endemic area of chronic arsenicism in Taiwan. J Natl Cancer Inst.

[3] Cebrian ME, Albores A, Aguilar M, Blakely E (1983). Chronic arsenic poisoning in the north of Mexico. Hum Toxicol.

[4] Vahidnia A, van der Straaten RJ, Romijn F, van Pelt J, van der Voet GB (2007). Arsenic metabolites affect expression of the neurofilament and tau genes: an in-vitro study into the mechanism of arsenic neurotoxicity. Toxicol In Vitro.

[5] Jolliffe DM, Budd AJ, Gwilt DJ (1991). Massive acute arsenic poisoning. Anesthesia.

[6] Pershagen G, Flower BA (1983). The epidemiology of human arsenic exposure. Biological and environmental effects of arsenic.

[7] He W, Greenwell RJ, Brooks DM, Calderon-Garciduenas L, Beall HD (2007). Arsenic exposure in pregnant mice disrupts placental vasculogenesis and causes spontaneous abortion. Toxicol Sci.

[8] Hill DS, Wlodarczyk BJ, Finnell RH (2008). Reproductive consequences of oral arsenate exposure during pregnancy in a mouse model. Birth Defects Res B Dev Reprod Toxicol.

[9] Sanghamitra S, Hazra J, Upadhyay SN, Singh RK, Amal RC (2008). Arsenic induced toxicity on testicular tissue of mice. Indian J Physiol Pharmacol.

[10] Santra A, Maiti A, Das S, Lahiri S, Charkaborty SK (2000). Hepatic damage caused by chronic arsenic toxicity in experimental animals. Toxicol Clin Toxicol.

[11] Gora RH, Baxla SL, Kerketta P, Patnaik S, Roy BK (2014). Hepatoprotective activity of Tephrosia purpurea against arsenic induced toxicity in rats. Indian J Pharmacol.

[12] Zaldívar R (1980). A morbid condition involving cardio-vascular, broncho-thoracic, digestive and neural lesions in children and young adults after dietary arsenic exposure. Zentralbl Bakteriol B.

[13] Karim MR, Haque A, Islam K, Ali N, Salam KA (2010). Protective effects of the dietary supplementation of turmeric (Curcuma longa L.) on sodium arsenite-induced biochemical perturbation in mice. Bangladesh Med Res Counc Bull.

[14] Sheikh A, Yeasmin F, Agarwal S, Rahman M, Islam K (2014). Protective effects of Moringa oleifera Lam. leaves against arsenic-induced toxicity in mice. Asian Pac J Trop Biomed.

[15] Parrish AR, Zheng XH, Turney KD, Younis HS, Gandolfi AJ (1999). Enhanced transcription factor DNA binding and gene expression induced by arsenite or arsenate in renal slices. Toxicol Sci.

[16] Suzuki N, Naranmandura H, Hirano S, Suzuki KT (2008). Theoretical calculations and reaction analysis on the interaction of prevalent thioarsenicals with biorelevant thiol compounds. Chem Res Toxicol.

[17] Wang JP, Wang SL, Lin Q, Zhang L, Huang D (2009). Association of arsenic and kidney dysfunction in people with diabetes and validation of its effects in rats. Environ Int.

[18] Rosenberg HG (1974). Systemic arterial disease and chronic arsenicism in infants. Arch Pathol.

[19] Chen Y, Hakim ME, Parvez F, Islam T, Rahman AM (2006). Arsenic exposure from drinking-water and carotid artery intima-medial thickness in healthy young adults in Bangladesh. J Health Popul Nutr.

[20] Koehler Y, Luther EM, Meyer S, Schwerdtle T, Dringen R (2014). Uptake and toxicity of arsenite and arsenate in cultured brain astrocytes. J Trace Elem Med Biol.

[21] Ding X, Su Q, Jiang M, Xie H, Cong J (2013). Arsenic affects on cerebellar development of mice. Toxicol Mech Methods.

[22] Rai NK, Ashok A, Rai A, Tripathi S, Nagar GK (2013). Exposure to As, Cd and Pb-mixture impairs myelin and axon development in rat brain, optic nerve and retina. Toxicol Appl Pharmacol.

[23] Kim M, Seo S, Sung K, Kim K (2014). Arsenic exposure in drinking water alters the dopamine system in the brains of C57BL/6 mice. Biol Trace Elem Res.

[24] Chandravanshi LP, Shukla RK, Sultana S, Pant AB, Khanna VK (2014). Early life arsenic exposure and brain dopaminergic alterations in rats. Int J Dev Neurosci.

[25] Ghosh A, Mandal AK, Sarkar S, Das N (2011). Hepatoprotective and neuroprotective activity of liposomal quercetin in combating chronic arsenic induced oxidative damage in liver and brain of rats. Drug Deliv.

[26] Islam K, Haque A, Karim R, Fajol A, Hossain E (2011). Dose-response relationship between arsenic exposure and the serum enzymes for liver function tests in the individuals exposed to arsenic: a cross sectional study in Bangladesh. Environ Health.

[27] Guha Mazumder DN (2005). Effect of chronic intake of arsenic-contaminated water on liver. Toxicol Appl Pharmacol.

[28] Liu DN, Lu XZ, Li BL, Zhou DX, Li FX (1992). Clinical analysis of 535 cases of chronic arsenic poisoning from coal burning. Chin J Med.

[29] Arteel GE, Guo L, Schlierf T, Beier JI, Kaiser JP (2008). Subhepatotoxic exposure to arsenic enhances lipopolysaccharide-induced liver injury in mice. Toxicol Appl Pharmacol.

[30] Sarkar S, Mukherjee S, Chattopadhyay A, Bhattacharya S (2014). Low dose of arsenic trioxide triggers oxidative stress in zebrafish brain: expression of antioxidant genes. Ecotoxicol Environ Saf.

[31] Okoji RS, Yu RC, Maronpot RR, Froines JR (2002). Sodium arsenite administration via drinking water increases genome-wide and Ha-ras DNA hypomethylation in methyl-deficient C57BL/6J mice. Carcinogenesis.

[32] Chen H, Li S, Liu J, Diwan BA, Barrett JC (2004). Chronic inorganic arsenic exposure induces hepatic global and individual gene hypomethylation: implications for arsenic hepatocarcinogenesis. Carcinogenesis.

[33] Xie Yaxiong, Liu Jie, Benbrahim-Tallaa Lamia, Ward Jerry M, Logsdon Daniel (2007). Aberrant DNA methylation and gene expression in livers of newborn mice transplacentally exposed to a hepatocarcinogenic dose of inorganic arsenic. Toxicology.

[34] Reichard JF, Schnekenburger M, Puga A (2007). Long term low-dose arsenic exposure induces loss of DNA methylation. Biochem Biophys Res Commun.

[35] Pant Niraj, Kumar Rakesh, Murthy Ramesh C, Srivastava Satya P (2001). Male reproductive effect of arsenic in mice. Biometals.

